# SCEDS: protein fragments for molecular replacement in *Phaser*

**DOI:** 10.1107/S0907444913021811

**Published:** 2013-10-04

**Authors:** Airlie J. McCoy, Robert A. Nicholls, Thomas R. Schneider

**Affiliations:** a Cambridge Institute for Medical Research, Department of Haematology, University of Cambridge, Hills Road, Cambridge CB2 0XY, England; b MRC Laboratory of Molecular Biology, Francis Crick Avenue, Cambridge Biomedical Campus, Cambridge CB2 0QH, England; c European Molecular Biology Laboratory, Hamburg Unit c/o DESY, Notkestrasse 85, 22603 Hamburg, Germany

**Keywords:** difference distance matrix, normal-mode analysis

## Abstract

A method is described for generating protein fragments suitable for use as molecular-replacement (MR) template models. The template model for a protein suspected to undergo a conformational change is perturbed along combinations of low-frequency normal modes of the elastic network model. The unperturbed structure is then compared with each perturbed structure in turn and the structurally invariant regions are identified by analysing the difference distance matrix. These fragments are scored with SCEDS, which is a combined measure of the sphericity of the fragments, the continuity of the fragments with respect to the polypeptide chain, the equality in number of atoms in the fragments and the density of C^α^ atoms in the triaxial ellipsoid of the fragment extents. The fragment divisions with the highest SCEDS are then used as separate template models for MR. Test cases show that where the protein contains fragments that undergo a change in juxtaposition between template model and target, SCEDS can identify fragments that lead to a lower *R* factor after ten cycles of all-atom refinement with *REFMAC*5 than the original template structure. The method has been implemented in the software *Phaser*.

## Abstract 2

Protein fragments suitable for use in molecular replacement can be generated by normal-mode perturbation, analysis of the difference distance matrix of the original *versus* normal-mode perturbed structures, and SCEDS, a score that measures the sphericity, continuity, equality and density of the resulting fragments.

## 1. Introduction

Conformational change in proteins ranges from a subtle change in residue positions, such as the loop switching in the Ras family of proteins (Vetter & Wittinghofer, 2001 ▶), to complete reorganization of multi-domain complexes, such as that observed in F_O_F_1_-ATP synthase (Okuno *et al.*, 2011 ▶). Conformational change defeats molecular replacement (MR; Rossmann & Blow, 1962 ▶) because it results in a high root-mean-square deviation (r.m.s.d.) in atomic positions between template structures (*i.e.* the MR search models) and target structures (*i.e.* the final successfully refined protein structures to be determined). The conformational changes of interest for this study are those involving a change in juxtaposition of fragments, where fragments are defined as spatially contiguous groupings of atoms for which all interatomic distances are effectively identical between the two conformations in the target and template, and for which the fraction of scattering is significant. These fragments may or may not correspond to definitions of protein domains such as those annotated in sequence databases such as ProDom (Servant *et al.*, 2002 ▶). This type of conformational change is interesting because, in principle, MR can be rescued by identifying the fragments in the template and using these as separate search models, hopefully, after positioning of the search models, replicating the change in juxtaposition of the fragments in the target.

It has been shown that conformational change in proteins can be modelled by normal-mode analysis (NMA) of the elastic network model (ENM; Bahar *et al.*, 1997 ▶, 1998 ▶; Haliloglu *et al.*, 1997 ▶; Haliloglu & Bahar, 1999 ▶; Tirion, 1996 ▶; Tama & Sanejouand, 2001 ▶; Krebs *et al.*, 2002 ▶). The ENM describes the mobility of the protein in terms of springs between neighbouring atoms and thereby models the local packing density of the protein. The first six normal modes of the interaction network are the three rigid-body rotations and the three rigid-body translations. The lowest mode of interest in the description of conformational change is therefore mode 7. One or more normal modes may contribute to a given conformational change (Tama & Sanejouand, 2001 ▶; Krebs *et al.*, 2002 ▶).

Suhre and Sanejouand successfully used deviations along normal modes to find MR solutions with *AMoRe* (Navaza, 1994 ▶) for maltodextrin-binding protein, HIV-1 protease and glutamine-binding protein where MR with *AMoRe* using the original templates failed (Suhre & Sanejouand, 2004 ▶). Because neither the normal modes nor normal-mode combinations that model the conformational change nor the distance of perturbation along the modes can be known *a priori*, template models for MR need to be generated for a number of low-frequency normal modes and combinations of normal modes and with a range of distances of perturbation in order to sample conformational space widely enough to spawn a template model with a sufficiently accurate conformation for MR to succeed.

As an alternative to modelling the conformational change, if the fragments can be identified in the template structure then MR can be performed with the fragments separately (Long *et al.*, 2008 ▶). The disadvantage of this approach lies in the cost that the fraction of the asymmetric unit being searched for with each fragment is smaller than the whole structure. The maximum-likelihood MR functions implemented in *Phaser* (McCoy *et al.*, 2007 ▶) account for the low fraction of scattering of search models that are only small components of the asymmetric unit and are sensitive to the placement of these partial models. The maximum-likelihood functions also use the information from already positioned components of the asymmetric unit extremely effectively by statistically accounting for the known component and its errors. The signal in MR searches for second and subsequent components of the asymmetric unit is greatly increased in the presence of the first (Read, 2001 ▶). With maximum-likelihood MR functions it is thus attractive to develop MR methods that utilize methods of performing MR that break the protein template into fragments, each with a low r.m.s.d. to its corresponding target fragment.

When the structure of a protein that undergoes a conformational change has been solved in two conformers, the structurally invariant regions can be identified (Wriggers & Schulten, 1997 ▶; Hayward & Berendsen, 1998 ▶; Abyzov *et al.*, 2010 ▶). Difference distance matrix (DDM) analysis has been deployed for this purpose in a number of studies (Nishikawa & Ooi, 1974 ▶; Nichols *et al.*, 1995 ▶; Schneider, 2000 ▶, 2002 ▶). A more challenging problem is the detection of structurally invariant regions of proteins for which the structure of only one conformer has been solved. Wodak and Janin were amongst the first to address this problem with their work on calculating surface area (Wodak & Janin, 1980 ▶). The *DynDom* method, developed for comparing two known structures, has been applied to the displacements generated by molecular-dynamics simulations (Hayward *et al.*, 1997 ▶; Hayward & Berendsen, 1998 ▶). The *TLSMD* method analyzes the distribution of anisotropic or isotropic *B* factors in refined protein crystal structures to generate optimal multi-group TLS descriptions of the protein (Painter & Merritt, 2006 ▶) similar to domain divisions (Zucker *et al.*, 2010 ▶). Normal-mode analysis was first used for identification of quasi-rigid domains by analysing the energy density as a function of position (Hinsen, 1998 ▶). *ProFlex* considers the rotatable and nonrotatable bonds of a protein and thus concerns the interactions stabilizing the protein similarly to NMA of the ENM (Jacobs *et al.*, 2001 ▶). *FlexOracle* calculates the free energy of folding for protein fragments based on all possible locations of one or two cuts in the polypeptide chain and thus is tailored to finding single-stranded or double-stranded hinges (Flores & Gerstein, 2007 ▶). The *DomDecomp* method determines domains using the sign of the Gaussian Network Model (GNM) normal mode 7 displacement and subsequent analysis that parallels graph-theory implementations (Kundu *et al.*, 2004 ▶). *StoneHinge* is built upon the *DomDecomp* and *ProFlex* algorithms (Keating *et al.*, 2009 ▶). *Hingemaster* is built upon *FlexOracle*, *TLSMD* and *ProFlex* with the addition of five normal-mode-based hinge-prediction algorithms (Flores *et al.*, 2008 ▶). These methods have their strengths and weaknesses in finding domain boundaries that depend on the underlying physical basis of the analyses (Flores *et al.*, 2008 ▶) and their CPU runtimes vary from minutes to tens of hours.

Historically, and currently in practice, fragments for use in MR are commonly identified by visual inspection of the template structure by the crystallographer, and this is still a gold standard for fragment identification despite the progress in automated methods. With training, it is possible to look at a structure on a graphics display and see (i) where the packing density of atoms is low and hence where flexibility is likely to occur, (ii) where the resulting fragments are roughly the same size or at least of significant size and so represent a significant fraction of the scattering and (iii) where the atoms are packed into contiguous regions of space; hence, where to divide the structure for MR. In this study, we show that it is possible to predict fragments of proteins suitable for MR using a combination of NMA, analysis of the DDM and a function that scores the size, shape and packing density of the fragments (SCEDS). The method is conceptually similar to the way that crystallographers identify fragments for MR from visual inspection of a structure.

## 2. Method

**Figure 1:**
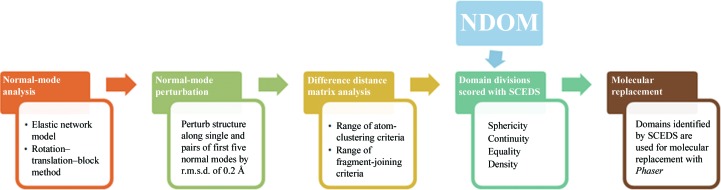
Flow diagram for SCEDS fragment analysis.

The SCEDS method for finding fragments for use in MR consists of five steps: (i) NMA of the ENM of the template structure, (ii) perturbation of the template structure along low-frequency normal modes of the ENM, (iii) using the DDM between the original and perturbed template structure to find the structurally invariant fragments for each perturbation, (iv) ranking of the structurally invariant fragments generated by each DDM analysis by SCEDS (the **S**phericity **C**ontinuity **E**quality **D**ensity **S**core) and (v) MR with the highest scoring fragments. Only one set of fragments representing the template, *i.e.* the set corresponding to the highest SCEDS, is used in MR at the conclusion of the analysis (Fig. 1 ▶).

The SCEDS method divides the protein into NDOM fragments as requested by the user, where NDOM is normally a small integer number (six or less) and is two by default. If the conformational change is more than a simple hinge motion then it will be necessary to generate fragment divisions for NDOM values higher than two in order for MR to succeed. SCEDS values are not comparable for analyses with different NDOM fragments and therefore the SCEDS method cannot determine an optimal number of fragments. The coordinates for the fragments written for MR searches are those of the unperturbed structure, because this structure has the unperturbed geometry.

### 2.1. NMA of the ENM

The ENM for the protein was implemented with a cutoff of 5 Å following the methods of Tirion (1996 ▶) and Bahar *et al.* (1997 ▶). The normal modes of the ENM are obtained by eigenvalue decomposition of the Hessian matrix, the 3*N* × 3*N* matrix of second derivatives of the energy with respect to the three spatial coordinates of the *N* atoms under consideration. For all but the smallest proteins, eigenvalue decomposition of this Hessian for all atoms is not computationally feasible. The rotation–translation block (RTB) approach (Durand *et al.*, 1994 ▶) implemented in *Phaser* projects the full Hessian into a lower dimension for decomposition. After eigenvalue decomposition of the projected Hessian, the motions of the atoms for each mode are found by de-projecting the corresponding eigenvectors. While this method is not suitable for modelling the high-frequency normal modes, it is able to model the lowest frequency normal modes that are the modes of interest for this study. Options for all-atom or C^α^-only Hessians are also implemented in *Phaser*. See the Supplementary Material^1^ for the parameters of the ENM.

### 2.2. Coordinate perturbation

After NMA, *n* normal modes can be used to generate 2^*n*^ − 1 combinations of normal modes. The number *n* of modes considered and the number of modes to combine (pairs, triplets and so on up to all *n* in unison) will influence the exploration of conformational differences between perturbed and unperturbed structures for the DDM analysis, and sufficient modes must be explored to include at least one combination that gives the correct fragments. At the same time, the computation time expands exponentially with the number *n* of normal modes if all combinations are considered. See the Supplementary Material for the parameters controlling the selection of normal modes and mode combinations.

### 2.3. DDM analysis

Only C^α^ atoms are included in the DDM. The DDM element (*a*, *b*) for a C^α^ atom with coordinates *U_a_* and *U_b_* in the unperturbed structure and coordinates *P_a_* and *P* *_b_* in the perturbed structure is 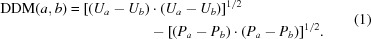



The DDM is analysed with a modified form of single-linkage clustering. C^α^ atoms *a* and *b* are considered for clustering if DDM(*a*, *b*) is less than a given threshold. Since the absolute numerical range of the values in the DDM depends on the degree of perturbation, the threshold depends on the range of DDM values present in the matrix. In addition to the difference distance threshold, two further tests must be passed before atoms *a* and *b* are clustered. Firstly, the atoms must be separated by less than a given distance through space, which helps to filter noise in the DDM. Secondly, a minimal separation of the atoms in sequence may be applied, preventing neighbouring atoms from being clustered together and thus the undesirable clustering of atoms through linker regions between fragments. The clustering of atoms in the DDM analysis is performed for a range of DDM thresholds, distances and sequence separations. Each choice of parameters has the potential to generate different sets of DDM clusters of atoms.

The algorithm for assigning the fragment number to each atom (or, viewed conversely, assigning atoms to each fragment) first compares atom *a* with atoms *b*→*z* and finds those meeting the three DDM-cluster criteria of DDM threshold, through-space distance and sequence separation. The atoms in the DDM cluster with *a* are assigned to fragment 1 by definition, with the remainder left unassigned. Atom *b* is then compared with atoms *c*→*z* and a set of DDM-cluster atoms are identified. This DDM cluster is assigned the fragment number of *b* (1) if *b* were previously clustered with *a* or if any member of the clustered subset of *c*→*z* were previously clustered with *a*. Otherwise, the DDM cluster of *b* will be assigned to fragment 2 by definition. Atom *c* is then compared with *d*→*z* to identify its DDM cluster. If neither *c* nor any members of its DDM cluster are assigned to a fragment this DDM cluster becomes fragment 3 by definition. If not, the DDM cluster is assigned the fragment number most represented in the DDM cluster. The algorithm iterates for the DDM cluster of *d* in *e*→*z* and so on until all atoms are assigned a fragment number.

This method of assigning C^α^ atoms to DDM clusters is dependent on the order in which the C^α^ atoms are considered, *i.e.* the sequence order. Whilst other modifications to single-linkage clustering that are independent of sequence order could be tried in the future, the SCEDS method implemented is very fast and produced good results in our test cases.

Note that, *via* the chaining of atoms together in fragments, the DDM-cluster through-space distance criterion does not limit the final spatial extent of the fragments and at the end of the analysis a fragment may fill any extent in space. Similarly, application of the DDM-cluster sequence-separation criterion does not force fragments to contain sequence gaps.

After the DDM analysis a fragment-joining procedure is undertaken. If fragment *m* contains a short intercalating fragment assigned to another fragment, the atoms in the fragment are assigned to fragment *m*. Fragment divisions are produced for a range of intercalating fragment-joining lengths.

At the end of the DDM analysis the fragments are sorted in size by the number of atoms assigned to each fragment. The sorted order will not (unless coincidentally) be the same as the integer order of the fragments because the latter results from the sequential sampling of the polypeptide chain for seed residues; fragment 1 will not generally be the largest fragment. For illustration, consider a protein with 100 residues, with the N-­terminal 39 and C-terminal 60 residues being folded fragments with a single-residue hinge between them at residue 40. Residue 1 would be in fragment 1 by definition. If NMA perturbation and DDM analysis revealed the hinge at residue 40, residues 2–39 would also be assigned to fragment 1, the hinge would be fragment 2 (with one residue) and the largest fragment, with 60 residues, would be fragment 3. The sort order for the fragments would be (3, 1, 2). After sorting the fragments by size, only the largest NDOM fragments are carried forward in the analysis and used in the SCEDS calculation. For the illustrative example above and NDOM = 2, this would be fragments 3 and 1.

The DDM analysis does not restrict the number of clusters of atoms to the number NDOM requested by the user. An important corollary is that the method does not guarantee that all atoms will be allocated to one or other of the top NDOM fragments. It is not valid to use these excluded atoms as an additional single fragment because the excluded atoms will have been assigned to multiple fragments.

At the conclusion of the DDM analysis step, some combinations of normal-mode perturbations and DDM analyses will result in the protein being divided into several large fragments of atoms while others will fragment the protein into many small fragments, depending on the nature of the flexibility revealed.

### 2.4. SCEDS

For the largest NDOM fragments from each perturbation and analysis of the DDM, a SCEDS is calculated. The SCEDS is the sum of four scores measuring the sphericity (*S*) of the fragments based on the maximal extents of the fragments in three orthogonal directions, the continuity (*C*) of the chains in the polypeptide sequence, the equality (*E*) in the number of amino acids in each fragment and the packing density (*D*) of the atoms in the fragments. Each of the four scores has a weight factor (*w*_*S*_, *w*_*C*_, *w*_*E*_ and *w*_*D*_, respectively),
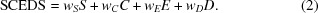
There may be some correlation between the four terms. Where the protein is built from NDOM equally sized and roughly spherical fragments, the equality and sphericity terms will be correlated. This will tend to give high SCEDS values precisely for the cases for which the algorithm is designed to favour. It is the ranking of the SCEDS values for the potential fragment divisions that is important, rather than their absolute value.

#### 2.4.1. Sphericity

Sphericity is calculated with the formula originally defined by Wadell (1935 ▶) for analysing the properties of quartz particles, but subsequently borrowed by other fields. It is a dimensionless measure of the volume (*V*_*d*_) to surface area (*A*_*d*_) ratio of the fragment *d*.
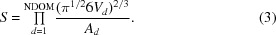
For the purposes of this study, the protein is modelled as a triaxial ellipsoid with principal axes *a*_*d*_, *b*_*d*_ and *c*_*d*_ of the fragment *d*. Although the volume of an ellipsoid is trivially calculated from the three principal axes, there is no trivial expression for the surface area, but an approximation (Thomsens’ formula; Thomsen, 2004 ▶) that yields values with a relative error of at most 1.061% is more than sufficient for this study,
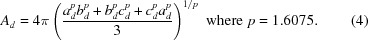
The sphericity has a maximum of 1 when the axes are equal.

#### 2.4.2. Continuity

A continuity measure penalizes those fragment divisions that involve sections of noncontiguous polypeptide chains. For each break a penalty is introduced, 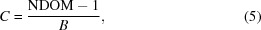
where *B* is the number of chain breaks in the proposed fragment division. *B* is at least one, this being the break between the first and second fragments. This function heavily penalizes the first chain break and then applies a less severe penalty for second and subsequent chain breaks. If there are no breaks within the fragments then the continuity term is 1.

#### 2.4.3. Equality

The equality is measured with the function
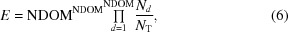
where *N*_T_ is the total number of C^α^ atoms in the protein and *N*_*d*_ is the number of C^α^ atoms in fragment *d* of the NDOM largest fragments. The equality reaches a maximum of 1 when there are NDOM equal-sized fragments that account for the whole of the structure with no gaps.

#### 2.4.4. Density

Fragments densely populated with atoms were selected by taking the product over the fragments of the packing of *N*_*d*_ C^α^ atoms into the volume defined by the triaxial ellipsoid used for the sphericity measurement for fragment *d*, 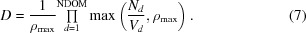
The maximal density ρ_max_ of C^α^ atoms in a structure was calculated from a test set of proteins looking at the density in spheres centred on the centre of mass of the proteins (data not shown) and is set at 0.0071 C^α^ Å^−3^. The density term has a maximal value of 1 when the triaxial ellipsoid is tightly packed with C^α^ atoms.

### 2.5. MR with SCEDS fragments

The fragments output from the SCEDS analysis are used for MR in *Phaser* using a standard MR protocol. The NDOM fragments are entered as NDOM separate ensembles. Assuming that the SCEDS fragment division is accurate, the r.m.s.d. can be accurately estimated from the sequence identity between the template and target in the same way as for a protein that does not have a conformational change between template and target (Oeffner *et al.*, 2013 ▶). The number of search copies entered into *Phaser* should be the same for each fragment, as the fragment segments are covalently linked, but the number of copies of each set of fragments will depend on the solvent content and the presence of any other components in the asymmetric unit, as indicated by the improved Matthews coefficient (Kantardjieff & Rupp, 2003 ▶) implemented in the *Phaser* cell-content analysis (CCA) mode. If MR is successful then structure refinement (*e.g.* with *REFMAC*; Murshudov *et al.*, 2011 ▶) and manual and/or automated building (*e.g.* with *Buccaneer*; Cowtan, 2006 ▶) of segments of the target structure that are incorrectly positioned or not included in the template structure should be alternated until all features of the electron density are explained. Recent methods of model building and refinement that include techniques drawn from *ab initio* modelling (DiMaio *et al.*, 2011 ▶) or morphing (Terwilliger *et al.*, 2012 ▶) increase the radius of convergence for automated building and refinement. The time required to complete structure refinement after MR with SCEDS depends on the quality of the phases from MR, which in turn depends on the completeness and accuracy of the models, as is the case for all MR.

For comparing different starting models after MR, a relative indication of how time-consuming refinement will be can be given from an initial round of automated refinement. Ten cycles of *REFMAC* (Murshudov *et al.*, 2011 ▶) are used for this purpose in this study. These cycles of refinement briefly optimize the input coordinates, but also, importantly, the *B* factors and the solvent parameters, and so give a useful crystallo­graphic *R* value. The lower the *R* value at the end of this initial step the more straightforward structure refinement will be.

## 3. Default parameters

Default weights for sphericity, continuity, equality and density terms in SCEDS, default selection of normal modes for normal-mode perturbations after NMA and default parameter ranges for the DDM analysis were found using a training set of proteins. To identify suitable defaults, parameters were sampled on a grid of values and the output for the cases in the training set were inspected to determine whether the known fragment divisions for these proteins had been obtained and to obtain the elapsed CPU times. The values identified as suitable defaults are a compromise between sampling possible fragment divisions widely enough to obtain correct fragment divisions in the training set whilst not consuming excessive CPU resources. Default parameters for the DDM analysis are set generously and the fragment divisions obtained are not significantly changed by some reduction in the range of parameters used. Changing the selection or combinations of normal modes and/or the weights of the terms in the SCEDS from the defaults will have a significant effect on the fragment divisions obtained. Using the defaults, the computation time is of the order of minutes for the proteins of the training set. This computation time is comparable to the time for the MR itself. Elapsed time is reduced by the use of shared memory multiprocessing (parallelization) in the implementation. CPU time for the analyses of a template with different values of NDOM may be shortened by reading in the eigenvector matrix written in the first analysis. The default parameters are described in the sections below and in the Supplementary Material.

### 3.1. Training set

The training-set proteins were selected on the basis that two conformers were present in the Protein Data Bank and the difference between the two conformers was a hinge motion between significant fractions of the polypeptide chains. There were ten proteins in the training set: alcohol dehydrogenase, cAMP-dependent protein kinase, citrate synthase, diptheria toxin, glutamine-binding protein, immunoglobulin, lactoferrin, lysine/arginine/ornithine (LAO) binding protein, maltodextrin-binding protein and thymine synthase. Three proteins were analysed in two conformers, giving 13 test cases (Table 1 ▶).

### 3.2. Perturbation of coordinates

Krebs and coworkers showed how 3814 cases of conformational change in proteins could be modelled with NMA (Krebs *et al.*, 2002 ▶). Most conformational changes could be modelled with between one and three low-frequency normal modes, with about half predominantly modelled with two modes. In 30% of cases the most predominant mode was mode 7, in 20% of cases it was mode 8 and in a further 25% of cases it was one of modes 9, 10 or 11. Based on this analysis, modes 7 to 11 are used alone or in combinations of two modes. This study thus uses more normal modes in the analysis than that of Kundu *et al.* (2004 ▶), which only uses mode 7. See the Supplementary Material for the parameters controlling the perturbation of coordinates.

By default, coordinates are perturbed in the positive and negative directions along the normal modes. Structures are generated per normal-mode combination with an r.m.s. deviation from the template of 0.2 Å. The displacement distance is not critical to SCEDS as it mostly just scales the DDM, and the DDM analysis is in turn scaled to the range of values in the matrix (see below) DDM analysis

By default, the range of DDM element thresholds sampled for clustering atoms together in a DDM cluster increases in five steps of a 50th of the range of values in the DDM from the minimum DDM element. The distance threshold ranges from 7 to 14 Å inclusive in steps of 1 Å and the separation threshold is either 0, or at least one trace atom. In total, 80 (5 × 8 × 2) DDM analyses are performed per normal-mode perturbation. Fragment-joining lengths range from 2 to 12% of the total polypeptide length. See the Supplementary Material for parameters controlling the DDM analysis.

### 3.3. SCEDS weights

Equality and density terms in SCEDS have weight factors of 1 by default. Sphericity is up-weighted with *w_S_* equal to 4 by default. The continuity weight *w_C_* is zero by default as nonzero values give poorer results in the majority of cases. This is likely to be because the N-­terminus and the C-­terminus of proteins are often located near one another and hence in the same fragment, and whole fragments can be inserted into loops (Thornton & Sibanda, 1983 ▶). Adding the continuity term to the analysis by giving a nonzero continuity weight (*w_C_*) will significantly change the SCEDS of fragment divisions and result in markedly different fragment divisions scoring highly. See the Supplementary Material for the parameters controlling SCEDS weighting.

## 4. Results

### 4.1. Test cases

The test cases for MR were selected on the basis that the protein had at least two conformations present in the Protein Data Bank and that at least one of these had structure factors available from the Uppsala Electron Density Server (Kleywegt *et al.*, 2004 ▶). The ten test cases involved seven proteins: adenylate kinase, cAMP-dependent protein kinase, calmodulin, citrate synthase, glutamine-binding protein, maltodextrin-binding protein and pyruvate phosphate dikinase (Table 1 ▶). Four of the ten protein conformers included in the test set were the open/closed conformation of the closed/open conformations included in the training set. These proteins were cAMP-dependent protein kinase, citrate synthase, glutamine-binding protein and maltodextrin-binding protein. The results from the test set may therefore be biased by the inclusion of some proteins of the same sequence in the training set. Mitigating this is the fact that NMA is dependent on the conformation of the protein rather than the sequence. It has been shown that open and closed conformations of proteins have very different normal-mode characteristics and that it is more difficult to predict the conformational change from the closed conformation than from the open conformation (Tama & Sanejouand, 2001 ▶).

### 4.2. Molecular replacement

MR searches were performed with *Phaser*. MR solutions were assessed by the *R* factor and *R*_free_ reported to 3 Å resolution after ten cycles of all-atom coordinate refinement in *REFMAC*5 (Murshudov *et al.*, 2011 ▶). As explained in §[Sec sec2.5]2.5, the lower the *R* factor after this step the more straightforward the refinement and structure completion following MR. If MR with the fragment divisions produced with NDOM = 2 failed to give MR solutions then NDOM was increased until a solution was found, up to a maximum of NDOM = 6.

**Figure 2:**
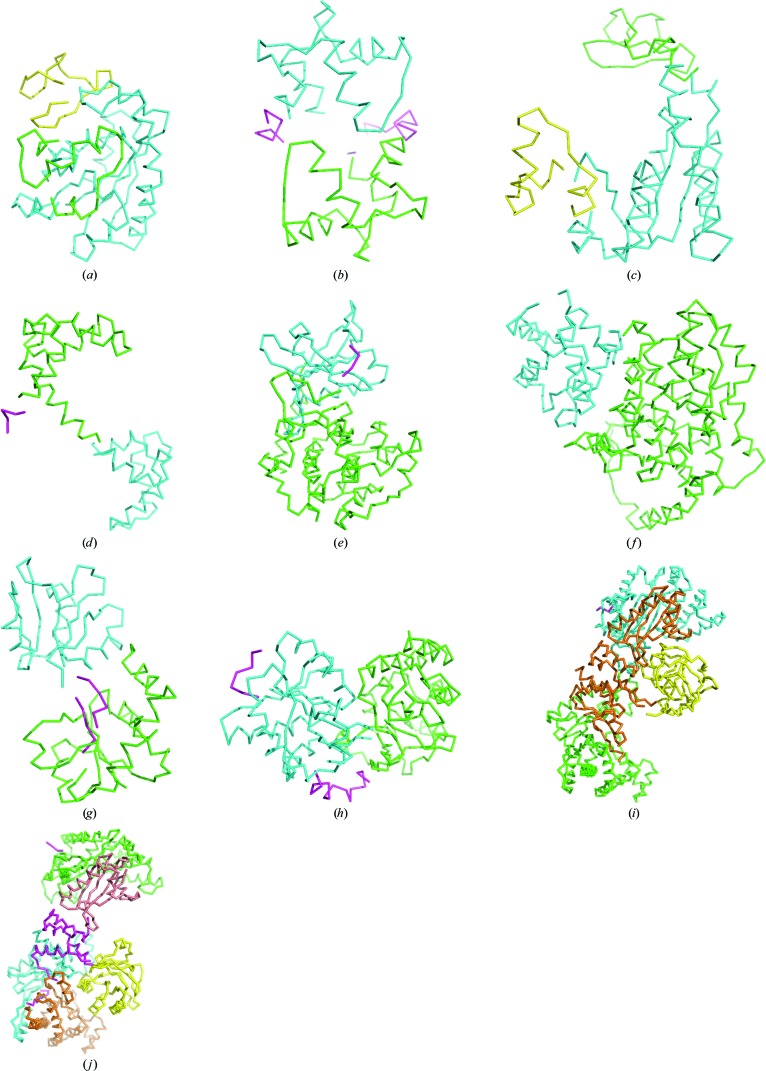
Ribbon representation of the SCEDS fragments for the ten cases in the test set coloured by fragment number, sorted on number of residues. The residues in each fragment are listed in Table 3 ▶. Fragment 1, green; fragment 2, blue; fragment 3, yellow; fragment 4, orange; fragment 5, salmon; residues excluded from the top NDOM fragments, magenta. NDOM = 2 unless otherwise stated. (*a*) Adenylate kinase, PDB entry 2eck, chain *B*; NDOM = 3. (*b*) Adenylate kinase, PDB entry 4ake, chain *A*; NDOM = 3. (*c*) Calmodulin, PDB entry 1ctr. (*d*) Calmodulin, PDB entry 1cll. (*e*) cAMP-dependent protein kinase, PDB entry 1atp. (*f*) Citrate synthase, PDB entry 5cse. (*g*) Glutamine-binding protein, PDB entry 1ggg. (*h*) Maltose-binding protein, PDB entry 1anf. (*i*) Pyruvate phosphate dikinase, PDB entry 1kbl. (*j*) Pyruvate phosphate dikinase, PDB entry 2r82. The figures were prepared with *PyMOL* (DeLano, 2002 ▶).

The results of the MR trials in the ten test cases using the fragments identified using SCEDS are shown in Table 2 ▶ and the fragment divisions are shown in Fig. 2 ▶. Full MR solutions were found for eight of the ten test cases. Of those that succeeded, all but pyruvate phosphate dikinase succeeded with the fragment division produced with NDOM = 2 (Figs. 2 ▶ *c*–2 ▶ *g*). The conformational change in pyruvate phosphate dikinase is more complicated than a simple hinge motion between two fragments (Lim *et al.*, 2007 ▶). For the target active pyruvate phosphate dikinase (PDB entry 1kbl) the template model in the inactive form had to be analysed with NDOM = 4 (Fig. 2 ▶ *h*) and for the target inactive pyruvate phosphate dikinase triple mutant (PDB entry 2r82) the template model in the active form had to be analysed with NDOM = 5 (Fig. 2 ▶ *i*). Where MR succeeded, the fragment divisions compared well with the fragment divisions obtained from a comparison of the two experimental structures with *ESCET* (Schneider, 2002 ▶). The pyruvate phosphate dikinase structure (PDB entry 1kbr) was also the only member of the test set that gave fragment divisions with a significant fraction of the atoms excluded. Approximately 8% of the scattering matter was not included in the fragments used for MR. This resulted in higher *R* values than would be expected had all residues in the template been included and correctly placed (Table 2 ▶).

MR failed for adenylate kinase in the open conformation in the target based on SCEDS analysis of the closed conformation. MR was partially successful for adenylate kinase in the closed conformation in the target, where two copies (corresponding to the two structures in the asymmetric unit) of the largest fragment identified from the SCEDS analysis of the open conformation with NDOM = 3 could be unequivocally placed in the asymmetric unit but the smaller fragments could not. The conformational change in adenlyate kinase has been extensively studied (Blaszczyk *et al.*, 2001 ▶). From comparison of the crystal structure of the open and closed form, Blaszczyk *et al.* (2001 ▶) describe the protein as having three domains: the CORE fragment (residues 3–29, 64–116 and 160–212), the LID domain (residues 117–150) and the NMP-binding domain (residues 30–63). The failure of MR with adenylate kinase after SCEDS even though the CORE, LID and NMP-binding domains for adenylate kinase were obtained with NDOM = 3 (Figs. 2 ▶ *a* and 2 ▶ *b*) is owing to the r.m.s.d. between the template and target for each fragment remaining high in the context of the low scattering fraction of the fragments, the quality of the data and the size of the unit cell in this case.

These results compare well with the results of Suhre and Sanejouand, who used *AMoRe* and template models generated as a result of perturbations along low-frequency normal modes to find MR solutions for maltose-dextrin binding protein, glutamine-binding protein and HIV-1 protease (Suhre & Sanejouand, 2004 ▶). HIV-1 protease was excluded from this study because the problem solves trivially in *Phaser* using the whole structure as a template and the conformational change was not that of a hinge motion between two or more large fragments. Suhre and Sanejouand used mode 7 for the solution of maltodextrin-binding protein and modes 7 and 8 for the solution of glutamine-binding protein. In this study, a combination of modes 7 and 9 gave the best SCEDS for malto­dextrin binding protein although a very similar fragment division was generated by mode 7 alone, and mode 8 gave the best fragment division for glutamine-binding protein. Suhre & Sanejouand perturbed citrate synthase along three normal modes and lactoferrin along the first 11 normal modes but did not find a model that succeeded in finding a solution with *AMoRe*. The fragment divisions from SCEDS gave MR solutions with low *R* values for these two proteins.

## 5. Discussion

SCEDS should prove useful to those attempting MR with proteins expected to undergo conformational change. Even when conformational change is not expected, the failure of an initial MR attempt could prompt SCEDS analysis of the template structure. SCEDS analysis is performed using only a single reference structure and does not require the knowledge of multiple conformers in advance. The fragment-generation method is fast, requiring little additional computation time compared with the subsequent MR step. SCEDS does not require access to a fragment database like *DOMAK* or *BALBES* (Siddiqui & Barton, 1995 ▶; Long *et al.*, 2008 ▶) and thus does not rely on the databases to be updated and efficiently curated. SCEDS is not reliant on a web server such as *HingeMaster* (http://www.molmovdb.org/; Flores & Gerstein, 2007 ▶) and thus does not compromise any confidentiality of the model structure. As a useful by-product of implementing this method in *Phaser* it is also possible to output structures perturbed along normal modes and combinations of normal modes directly for use in MR as complete templates following the method of Suhre and Sanejouand (using *ElNémo*; Suhre & Sanejouand, 2004 ▶; see Supplementary Material).

In contrast to other studies of protein flexibility using NMA, the aim here is not to model the biological conformational change itself. The aim is simply to use the normal modes to identify fragments that are considered to be likely to maintain rigidity despite the particular conformational state/biological assembly of the protein, as this is of most use for MR. Our definition of a fragment is that it is a globular fragment of a protein that is sufficiently rigid and sizeable for MR to succeed. It follows that the normal modes that give a fragment division which results in successful MR may or may not correspond to the normal modes found to model the biological conformational change. When modelling the biological conformational change the direction of the motion between the fragments needs to be modelled in addition to the fragments being (implicitly) identified. We have observed that often several mode combinations sharing one or more normal modes can generate similar fragments. The movement between two biological conformations is stabilized *in vivo* by interaction of the protein with another protein or a small-molecule substrate. This study cannot include consideration of these specific interactions in the ENM since they are only known *post hoc*. Specific interactions may influence the specific normal modes that contribute to the direction of the conformational change (Dobbins *et al.*, 2008 ▶).

The test cases used here only include structures of the same sequence undergoing a conformational change. However, structures with lower sequence identity may also be subjected to this analysis and used in MR. This technique can be used in conjunction with model-editing procedures either before or after SCEDS analysis is undertaken (Schwarzenbacher *et al.*, 2004 ▶; Bunkóczi & Read, 2011 ▶; Vagin & Teplyakov, 2010 ▶).

The algorithm can be run with NDOM = 1. For the multi-fragment cases in this study, the single fragment output is either the whole protein with some trimming or the same as the largest fragment in the two-fragment analysis. The algorithm is biased towards assigning all atoms to the top NDOM fragments through the equality term. As currently implemented, SCEDS is therefore not suitable for locating flexible regions on the surfaces of proteins, and other loop-trimming procedures should be used for this purpose (Bunkóczi & Read, 2011 ▶).

In the future, it may be possible to design a method to automatically select the optimal number of fragments. The silhouette width, which measures how well DDM-clustered C^α^ atoms fit their DDM cluster (Rousseeuw, 1987 ▶), could contribute to deciding the best NDOM. The silhouette width could also be used to order the fragments by their separation rather than their size. However, keeping the total amount of scattering close to the total in the asymmetric unit is important to the success of the MR, and methods that favour compact well separated fragments but that result in the discarding of large fractions of the total scattering matter are not likely to give better MR results.

The SCEDS method is not infallible and fragments useful for MR may not be generated. This will do no harm when the technique is used for MR, because the MR itself acts as a positive control on the accuracy of the fragment divisions, failing if they are wrong and succeeding if they are correct. We would caution against using SCEDS fragments for any structural analysis other than MR.

## 6. Availability

All methods described are implemented in *Phaser*. *Phaser* is available through the *CCP*4 (http://www.ccp4.ac.uk; Winn *et al.*, 2011 ▶) and *PHENIX* (http://www.phenix-online.org; Adams *et al.*, 2002 ▶) software distributions. *Phaser* documentation can be found at http://www.phaser.cimr.cam.ac.uk.

## Supplementary Material

Supplementary material file. DOI: 10.1107/S0907444913021811/ba5209sup1.pdf

## Figures and Tables

**Table 1:** Structures used in SCEDS training and test sets Where there were multiple copies in the asymmetric unit, chain *A* was used for the SCEDS analysis.

	Target	Target	Template	Template
	PDB code	Conformer	PDB code	Conformer
Training set				
Alcohol dehydrogenase	6adh	Closed	8adh	Open
Alcohol dehydrogenase	8adh	Open	6adh	Closed
cAMP-dependent protein kinase	1atp	Open	1ctp	Closed
Citrate synthase	6csc	Closed	5csc	Open
Diptheria toxin	1mdt	Closed	1ddt	Open
Diptheria toxin	1ddt	Open	1mdt	Closed
Glutamine-binding protein	1wdn	Closed	1ggg	Open
Immunoglobulin	1hil, chain *B*	Unbound	1him, chain *M*	Bound
Lactoferrin	1flg	Closed	1flh	Open
Lactoferrin	1flh	Open	1flg	Closed
LAO binding protein	2lao	Open	1lst	Closed
Maltodextrin-binding protein	1anf	Closed	1omp	Open
Thymine synthase	2tsc	Closed	3tms	Open
Test set				
Adenylate kinase	1ake	Open	2eck	Closed
Adenylate kinase	2eck	Closed	1ake	Open
cAMP-dependent protein kinase	1ctp	Open	1atp	Closed
Calmodulin, closed	1ctr	Closed	1cll	Open
Calmodulin, open	1cll	Open	1ctr	Closed
Citrate synthase	6csc	Closed	5csc	Open
Glutamine-binding protein	1wdn	Closed	1ggg	Open
Maltodextrin-binding protein	1omp	Open	1anf	Closed
Pyruvate phosphate dikinase	1kbr	Active	2t82	Inactive
Pyruvate phosphate dikinase	2t82	Inactive	1kbr	Active

**Table 2:** *R* values/*R*_free_ after ten cycles of *REFMAC* (Murshudov *et al.*, 2011 ▶) for structures of the whole template superimposed on the target (template) and after MR with the template divided into SCEDS fragments using the value of NDOM given The conformational change for calmodulin between the closed and open forms is too large to allow the whole template in one conformation to be superimposed on the target in the other conformation. All *R* values/*R*_free_ values are for data to a high-resolution limit of 3 Å. Automatic local NCS restraints were used for the two copies of citrate synthase in the asymmetric unit. The two test cases for adenylate kinase are omitted because MR failed.

	*R* value (*R*_free_), template	*R* value (*R*_free_), fragments	NDOM
Calmodulin, closed	—	0.27 (0.34)	2
Calmodulin, open	—	0.28 (0.37)	2
cAMP-dependent protein kinase	0.36 (0.39)	0.28 (0.29)	2
Citrate synthase	0.35 (0.38)	0.26 (0.30)	2
Glutamine-binding protein	0.48 (0.47)	0.32 (0.39)	2
Maltodextrin-binding protein	0.42 (0.52)	0.22 (0.32)	2
Pyruvate phosphate dikinase	0.42 (0.47)	0.27 (0.35)	4
Pyruvate phosphate dikinase, triple mutant	0.42 (0.47)	0.29 (0.37)	5

**Table 3:** Fragments generated by SCEDS as shown in Fig. 2 ▶ MR succeeded in cases (*c*)–(*j*). For adenylate kinase cases (*a*) and (*b*) the SCEDS fragments listed as most closely resembling the CORE, LID and NMP-binding (NMP) domains (Blaszczyk *et al.*, 2001 ▶) are listed although MR with these failed.

	PDB code	Modes	NDOM	Fragment 1	Fragment 2	Fragment 3	Fragment 4	Fragment 5	Excluded
(*a*) Adenylate kinase, closed	2eck, chain *B*	7, 9	3	1–35, 68–124, 155–214 (CORE)	36–67 (NMP)	125–154 (LID)	—	—	None
(*b*) Adenylate kinase, open	4ake, chain *A*	7, 8	3	1–34, 68–117, 164–214 (CORE)	118–163 (LID)	35–67 (NMP)	—	—	None
(*c*) Calmodulin, closed	1ctr	9, 10	2	9–83	84–147	—	—	—	4–8
(*d*) Calmodulin, open	1cll	9, 10	2	12–72	88–147	—	—	—	1–11, 73–73, 81–87
(*e*) cAMP-dependent protein kinase	1atp, chain *E*	7, 8	2	15–35, 125–328	36–124, 222–350	—	—	—	329–332
(*f*) Citrate synthase	5csc, chain *A*	7, 8	2	1–82, 84–276, 385–433	277–291, 295–384	—	—	—	None
(*g*) Glutamine-binding protein	1ggg, chain *A*	8	2	5–82, 189–224	89–183	—	—	—	83–88, 184–188
(*h*) Maltodextrin-binding protein	1anf	7, 9	2	113–258, 319–370	8–112, 259–303	—	—	—	1–7
(*i*) Pyruvate phosphate dikinase	1kbl	11	4	566–828	6–243	244–380, 515–565, 829–873	381–514	—	2–5
(*j*) Pyruvate phosphate dikinase, triple mutant	2r82	7, 10	5	6–242	709–873	539–699	379–515	243–338	2–5, 340–370, 516–538, 700–708

## Footnotes

1 Supplementary material has been deposited in the IUCr electronic archive (Reference: BA5209). Services for accessing this material are described at the back of the journal.
