# Supplementary material for: SCEDS: protein fragments for molecular replacement in Phaser
Source: Acta Crystallogr D Biol Crystallogr. 2013 Oct 4;69(Pt 11):2216–25. doi: 10.1107/S0907444913021811 (PMC3817695; doi:10.1107/S0907444913021811)
Supplement: Supplementary file 1 [file d-69-02216-sup1.pdf]

Supplementary Material

This document describes the keywords for controlling SCEDS functionality in *Phaser*-2.5.5 (released 4 September 2013). Any changes made to the keywords controlling SCEDS functionality in subsequent versions of *Phaser* will be documented at the Phaserwiki http://www.phaser.cimr.cam.ac.uk.

The SCEDS functionality is accessed using MODE SCEDS. Coordinate perturbation along normal modes is accessed using MODE NMA.

Parameters for the Elastic Network Model

ENM RADIUS <RADIUS>

Defines the Elastic Network Model interaction radius. Default 5 Angstroms.

ENM FORCE <FORCE>

Defines the Elastic Network Model force constant. Default 1.

ENM OSCILLATORS [RTB | CA | ALL]

Defines the oscillators, either rotation-translation-blocks (RTB), C-alpha atoms (CA) or all-atom (ALL) for the decomposition of the Elastic Network Model. Default is RTB.

ENM MAXBLOCKS <MAXBLOCKS>

Defines the number of rotation-translation blocks for the RTB oscillators. This determines the trade-off between accuracy and speed of the calculation, with higher numbers of blocks giving finer grained sampling of the elastic network, but much higher computation times. Default 125.

ENM NRES <NRES>

Defines the number of residues in a block for the RTB method. By default, NRES is calculated so that it is as small as possible without reaching MAXBLOCKS blocks in total.

Parameters for the Mode Selection

NMA MODE <M>

Can be used multiple times to add multiple modes defining the mode(s) used for the perturbation of the coordinates. The first non-trivial mode is 7. A value M of less than 7 is taken to mean the first M modes starting at mode 7, so 5 gives modes 7 8 9 10 and 11. Default is 5.

NMA COMBINATION <NCOMB>

Defines the maximum number of modes taken in combination. Default is 2.

Parameters for the Coordinate Perturbations

PERTURB INCREMENT [RMS| DQ]

Perturb the structure by RMS increments along the modes (RMS), or by set dq increments (DQ). Default RMS.

PERTURB RMS STEP <RMS>

PERTURB RMS CLASH <CLASH>

PERTURB RMS STRETCH <STRETCH>

PERTURB RMS MAX <MAXRMS>

PERTURB RMS DIRECTION [FORWARD|BACKWARD|TOFRO]

If the structure is perturbed in RMSD increments, STEP (Default 0.2) defines increment in RMS Ångstroms between the template structure and the perturbed coordinates. The structure will be perturbed along each mode until either the C-alpha atoms come within CLASH (Default 2) Ångstroms of other C-alpha atoms, the distances between C-alpha atoms stretch a distance STRETCH (Default 5) Ångstroms too far (note that normal modes do not preserve the geometry) or until the MAXRMS (Default 0.3) Ångstroms RMSD has been reached. The structure can be perturbed either forwards or backwards or to-and-fro (FORWARD | BACKWARD | TOFRO) (Default TOFRO) along the eigenvectors of the modes specified.

PERTURB DQ <DQ1> *{DQ <DQ2>…}*

Alternatively, the DQ factors (as used by the Elnemo server (Suhre & Sanejouand, 2004) by which to perturb the atoms along the eigenvectors can be entered directly.

Parameters for DDM Analysis

DDM SLIDER <VAL>

The SLIDER window width is used to smooth the DDM. Default 0 (no smoothing).

DDM DISTANCE MIN <MIN> MAX <MAX> STEP <STEP>

Range and step interval for the DDM analysis. Range is from DDM value MIN/STEP to MAX/STEP. Default 1, 5 and 50 respectively.

DDM SEPARATION MIN <MIN> MAX <MAX>

Range for through space separation in Ångstroms. Default 7 and 14 respectively.

DDM SEQUENCE MIN <MIN> MAX <MAX>

Range for sequence separations between matrix pairs. Default 0 and 1 respectively.

DDM JOIN MIN <MIN> MAX <MAX>

Range for lengths of the sequences to join if fragment segments are discontinuous, in percentages of the polypeptide chain. Default 2 and 12 respectively.

Parameters for SCEDS

SCEDS NDOM <NDOM>

Number of fragments NDOM into which to split the protein. Default 2.

SCEDS WEIGHT SPHERICITY <WS>

Weight factor for Sphericity in SCEDS. The Sphericity Test scores boundaries that divide the protein into more spherical fragments more highly. Default 4.

SCEDS WEIGHT CONTINUITY <WC>

Weight factor for Continuity in SCEDS. The Continuity Test scores boundaries that divide the protein into fragments contiguous in sequence more highly. Default 0 (not used).

SCEDS WEIGHT EQUALITY <WE>

Weight factor for Equality in SCEDS. The Equality Test scores boundaries that divide the protein more equally more highly. Default 1.

SCEDS WEIGHT DENSITY <WD>

Weight factor for Density in SCEDS. The Density Test scores boundaries that divide the protein into fragments more densely packed with atoms more highly. Default 1.

Parameters for Input/Output

EIGEN WRITE [ON | OFF]

Write a file containing the eigenvalues and eigenvectors from the NMA of the ENM.

EIGEN READ <EIGENFILE>

Read a file containing the eigenvalues and eigenvectors from the NMA of the ENM written by a previous run of Phaser. The Phaser run that generated the eigenfile and the run reading the eigenfile must use identical ENM parameters.
